# Frequency of the necessity of dentoalveolar surgery or conservative treatment in patients before kidney transplantation depending on the duration of dialysis and causative nephrological disease

**DOI:** 10.1007/s00784-021-04202-1

**Published:** 2021-10-08

**Authors:** Tobias Moest, Rainer Lutz, Arne Eric Jahn, Katharina Heller, Mario Schiffer, Werner Adler, James Deschner, Manuel Weber, Marco Rainer Kesting

**Affiliations:** 1 Department of Oral and Maxillofacial Surgery, University Hospital Erlangen, Glueckstraße 11, 91054 Erlangen, Germany; 2 Department of Nephrology and Hypertension, University Hospital Erlangen, Erlangen, Germany; 3 Department of Medical Informatics, Biometry and Epidemiology (IMBE), University of Erlangen-Nuremberg, Erlangen, Germany; 4 Department of Periodontology and Operative Dentistry, University of Mainz, Mainz, Germany

**Keywords:** Oral health, Organ transplantation, Kidney, Immunosuppression

## Abstract

**Objectives:**

This retrospective study evaluates intraoral surgical and conservative treatment need in patients with a chronic kidney end-stage disease, depending on the duration of dialysis treatment and the causative nephrological disease.

**Material and methods:**

This study is based on data of patients referred to the Department of Oral and Maxillofacial Surgery of the University Hospital Erlangen, Germany, prior to kidney transplantation between January 2015 and March 2020. The necessity for oral surgical or dental therapy was determined by clinical and radiological examinations. Data on renal replacement therapy, cause of underlying renal disease, lifestyle, and general health were collected.

**Results:**

Data of *N* = 89 patients demonstrated that surgical treatment need depends on dialysis duration (*p* = 0.042). Patients, who had been dialyzing for 2 to 3 years showed the highest need for surgical intervention (80.0%; *p* = 0.024), followed by dialysis patients with a dialysis time of more than 3 years (48.1%). Similarly, dialysis patients in the second or third year of dialysis had the highest need for conservative treatment (73.3%; *p* > 0.05), followed by 55.6% of dialysis patients in the third year of dialysis.

**Conclusions:**

Operative and conservative treatment is essential to optimize subsequent kidney transplantation. The greatest necessity could be detected for patients in the second and third years of dialysis.

**Clinical relevance:**

Oral health addressing surgical and conservative treatment need depends on the duration of dialysis in patients with a chronic kidney end-stage disease.

## Introduction

Chronic renal disease with resulting terminal renal insufficiency represents a growing problem worldwide. Globally, more than two million people are being treated for end-stage renal disease [1]. Chronic renal disease is characterized by an impairment of the kidneys’ filtration and excretion function, leading to disturbances of the volume load and the electrolyte and acid-base balance. Furthermore, chronic renal insufficiency results in insufficient hormone production, consequently leading to anemia or osteopathy.

Frequently, chronic renal disease ultimately leads to terminal renal insufficiency, which is only treatable with renal replacement procedures, the most common of which are hemodialysis and allogeneic kidney transplantation. Due to the high burden of hemodialysis on the patient’s lifestyle and well-being and several serious symptoms associated with terminal kidney failure, the average life expectancy following initiation of hemodialysis is significantly impaired [2]. For long-term patient survival, organ transplantation as well as associated lifelong drug-induced immunosuppression are imperative.

There is ample evidence that patients suffering from chronic renal insufficiency commonly display poor oral hygiene and present with an insufficient oral health status [3–5]. In light of the well-established association between renal insufficiency under hemodialytic treatment and the occurrence, severity, and progress of infections like periodontal disease in such patients, the role of dentists and oral and maxillofacial surgeons in monitoring and treating this patient population is essential. The therapy of infection in the head and neck region in an immunosuppressed patient may necessitate the interruption of immunosuppressive medication in order to achieve immune competence for surgery, with a concomitant risk of organ rejection and consequently reduced patient survival. Therefore, the diagnosis and prophylactic treatment of existing infections, like destroyed teeth and residual roots prior to organ transplantation and drug-induced immunosuppression, are essential. In this context, studies underline that immunosuppressed patients are at greater risk of developing systemic infections leading to hospitalization [6] or even organ rejection relating to odontogenic infection [7]. Oral rehabilitation prior to kidney transplantation is therefore imperative.

In order to eliminate any dental or other foci for oral infection prior to organ transplantation, the Department of Nephrology and Hypertension of the University Hospital Erlangen, Germany, routinely refers patients with terminal renal insufficiency to the Department of Oral and Maxillofacial Surgery of University Hospital Erlangen, Germany, for clinical and radiological evaluation and treatment of present or potential intraoral infections. Patients suffering from renal insufficiency are already at high risk of intra- and postoperative complications, in many cases due to co-morbidities and medications prescribed for them, such as prescription anticoagulants or residual anticoagulant effects from hemodialysis and immunomodulatory medications. Thus, their management requires precise diagnosis, therapeutic planning, and postoperative care.

It is well established that patients suffering from chronic renal disease display a worse overall dental status compared to the general population, with a lower number of permanent teeth, and a higher number of decayed teeth [5, 8]. However, these well-documented shortcomings with regard to oral health present in this patient population do not directly reflect the need for surgical and/or conservative dental treatment. Nevertheless, the need of invasive or noninvasive dental treatment represents relevant information for the practitioner in order to estimate the possible treatment effort for such a patient, with a high-risk profile for intra- and postoperative complications.

This retrospective clinical observational study intends to assess the surgical and conservative therapeutic need in patients suffering from terminal renal insufficiency, who were referred to the Department of Oral and Maxillofacial Surgery of the University Hospital Erlangen prior to organ transplantation and subsequent drug-induced immunosuppression.

We assume as a null hypothesis that there are no differences concerning the need for surgical and/or conservative intervention between the different groups with regard to the etiology of terminal renal insufficiency, the duration of dialytic treatment, or the existence of additional risk factors (e.g., nicotine or alcohol abuse).

## Materials and methods

### Study characteristics

This retrospective clinical observational study was executed collaboratively by the Department of Nephrology and Hypertension and the Department of Oral and Maxillofacial Surgery at the University Hospital Erlangen, Germany. In the process of being listed as prospective recipients of a donor organ, patients suffering from terminal renal insufficiency were recruited by the Department of Nephrology and Hypertension and sent to the outpatient clinic of the Department of Oral and Maxillofacial Surgery of the University Hospital Erlangen for the evaluation of their intraoral health status and the initiation of appropriate surgical and conservative procedures prior to organ transplantation. The study encompasses an observational period from January 2015 to March 2020.

This study was approved by the ethical review board of the medical faculties at Friedrich-Alexander-University Erlangen-Nuremberg (Petition No. 450_20 Bc).

### Inclusion and exclusion criteria

Patients with terminal renal insufficiency who were referred to the outpatient clinic of the Department of Oral and Maxillofacial Surgery of the University Hospital Erlangen by the Department of Nephrology and Hypertension for clinical and radiological evaluation before organ transplantation were included in the study. Patients not yet receiving hemodialysis, as well as those patients who refused clinical and/or radiological assessment, were excluded from the study.

### Outcome variables

The primary outcome variable of the study was the quantification of the necessity for surgical intervention in patients with terminal renal insufficiency as a function of the duration of dialytic therapy, with the goal of either treating or preventing local inflammatory processes prior to organ transplantation.

The secondary outcome variable is an assessment of whether the causative nephropathy is associated with the necessity for surgical intervention.

### Assessment of oral status

All participants underwent a standardized oral examination by an experienced oral and/or maxillofacial surgeon. We assessed dental health status using the DMFT method. The DMFT index expresses the total number of decayed teeth (D-T), the total number of missing teeth (M-T), and the total number of filled teeth (F-T). Teeth with suspicious or readily apparent cavities were distributed to the D (= decayed) category. Missing teeth were distributed the M (= missing) category, and filled teeth were assigned to the F (= filled) category. Wisdom teeth were not taken into consideration. In addition, the clinical examination included an assessment of a tooth’s degree of loosening (graded at I–III) and its percussion sensitivity (+ or −). The periodontal status was assessed using a fixed periodontal probe (PCP 12, Hu-Friedy, Chicago, IL, USA).

In order to assess intraosseous lesions, a standardized orthopantomogram was performed (Sirona Orthophos XG; Sirona Dental Systems GmbH, Bensheim, Germany).

### Data collection

Gender, age, systemic (general) diseases, medications, allergies, as well as nicotine, alcohol, and/or illicit drug abuse were documented with the help of a standardized anamnesis questionnaire. Transplant-specific questions (e.g., type and duration of dialysis, cause of terminal renal insufficiency) were documented in consultation with the Department of Nephrology and Hypertension’s kidney transplant center. With regard to the duration of dialysis, we further divided the patient population into a “less than 2 years” group, a “2 to 3 years,” and a “more than 3 years” group.

### Determination of treatment need

The need for surgical and/or conservative intervention was based on the findings of the radiological and clinical examinations.

An indication for surgical treatment was assigned to deeply destroyed teeth, residual roots without any prosthodontic value, strongly loosened teeth (grade III of loosening), teeth with persistent or recurring periapical lesions after root canal treatment (e.g., radicular cyst after unsuccessful root canal treatment), and teeth with primary periapical osteolysis and cystic lesions. We introduced the subcategories of “no surgical intervention needed,” “one to two teeth should be removed,” “three to four teeth should be removed,” and “more than four teeth should be removed” to provide a more nuanced picture of the degree of severity with regard to the necessity for surgical intervention.

For teeth presenting with carious lesions, for non-vital teeth with positive percussion sensitivity, with or without apical lesions, and for teeth with periodontitis, a conservative treatment strategy was recommended.

### Statistical analysis

To analyze relationships between surgical treatment need and conservative treatment need with duration of dialysis, causative disease, categorized age, and alcohol and nicotine abuse, we performed Fisher’s exact tests. Multifactorial logistic regression models with conservative treatment need and surgical treatment need as dependent variables and age, diabetes as causative disease, duration of dialysis, and substance abuse as predictors were calculated, and odds ratios and corresponding 95% intervals were given. The significance level was set to 0.05. Statistical analysis was performed using the programming language R version 4.0.3 [9].

## Results

### Study population

Between January 2015 and March 2020, 111 patients were referred to the Department of Oral and Maxillofacial Surgery at the University Hospital Erlangen, Erlangen, Germany, by the Department of Nephrology and Hypertension for consultation, radiological/intraoral status evaluation, and initiation of necessary surgical/conservative procedures prior to organ transplantation. *N* = 22 (19.8%) patients were excluded from the study due to exclusion criteria (*N* = 20 (18.0%) no dialysis as of yet and *N* = 2 (1.8%) refused radiological/clinical examination) being fulfilled, and *N* = 89 patients were included in the study.

### Characterization of study population

According to the inclusion and exclusion criteria, *N* = 89 patients aged between 18 and 79 years (mean: 50.53 ± 13.00 years, median: 52 years) were included in the study. *N* = 20 (22.5%) patients were younger than 40 years, *N* = 47 (52.8%) patients were aged 40 to 59 years, and *N* = 22 (24.7%) patients were 60 years or older. *N* = 25 (28.1%) of included patients were female. In total, 86.5% of patients received hemodialysis and 13.5% of patients received peritoneal dialysis.

With regard to the duration of dialytic treatment, in *N* = 47 (52.8%) cases, patients had been receiving dialysis for less than 2 years, in *N* = 15 cases (16.9%) 2 to 3 years, and in *N* = 27 cases (30.3%) more than 3 years. In *N* = 5 cases, patients had received dialysis for longer than 10 years, one of which (*N* = 1) had received dialytic treatment for a total of 18 years.

Focusing on the reason for terminal renal insufficiency, *N* = 23 (25.8%; mean: 55.78 ± 11.77 years, median: 54 years; mean DMFT index: 22.04) patients had diabetic nephropathy, *N* = 14 (15.7%; mean: 51.29 ± 8.03 years, median: 53 years; mean DMFT index: 17.07) had an autosomal dominant polycystic kidney disease (ADPKD), *N* = 12 (13.5%; mean: 50.17 ± 14.55 years, median: 53 years; mean DMFT index: 15.58) received an unclear diagnosis/genesis, *N* = 9 (10.1%; mean: 51.33 ± 12.98 years, median: 53.0 years; mean DMFT index: 13.44) had an IgA nephropathy, *N* = 8 (9.0%; mean: 50.50 ± 15.49 years, median: 56.5 years; mean DMFT index: 12.00) were suffering from focal segmental glomerulosclerosis/sclerosing glomerulonephritis (FSGS), *N* = 5 (5.6%; mean 53.20 ± 7.33 years, median: 55 years; mean DMFT index: 17.20) had a hypertensive nephropathy, *N* = 5 (5.6%; mean: 36.00 ± 20.99 years, median: 32 years; mean DMFT index: 10.80) had vesicourethral reflux nephropathy, *N* = 4 (4.5%; mean: 40.25 ± 7.14 years, median: 38.5 years; mean DMFT index: 9.5) suffered from an atrophic (shrunk) kidney, *N* = 3 (3.4%; mean: 44.33 ± 5.51 years, median: 44.0 years; mean DMFT index: 15.00) suffered from amyloid nephropathy, *N* = 2 (2.2%; mean: 40.00 ± 1.41 years, median: 40.0 years; mean DMFT index: 10.00) had other forms of glomerulonephritis, *N* = 2 (2.2%; mean: 64.00 ± 16.97 years, median: 64.0 years; mean DMFT index: 16.00) had a nephropathy due to agenesis, *N* = 1 (1.1%; 55 years; DMFT index: 22) had minimal change glomerulonephritis, and *N* = 1 (1.1%; 34 years; DMFT index: 4) had a nephropathy due to hemolytic-uremic syndrome.

### Surgical treatment need depending on dialysis duration

**Fig. 1:**
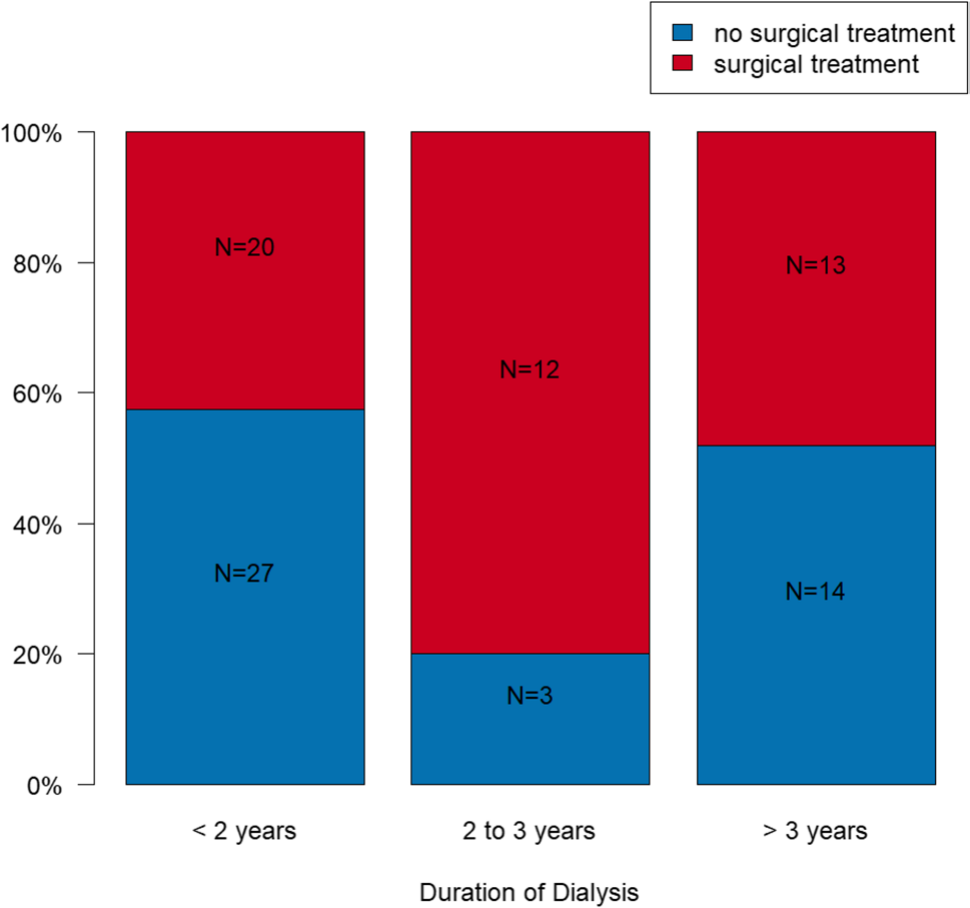
Surgical (red) and no surgical treatment (blue) need depending on duration of dialysis

For patients having been receiving dialysis for less than 2 years, in *N* = 27 cases (57.4%), no surgical intervention was necessary. In *N* = 13 patients (27.7%), the extraction of one or two teeth, in *N* = 3 cases (6.4%) of three or four teeth, and in *N* = 4 cases (8.5%) of more than four teeth was recommended. There were obvious differences between patients who had been receiving dialysis for 2 or 3 years when compared to patients with less than 2 years of dialytic treatment. Among the former group, no surgical intervention was necessary in merely *N* = 3 cases (20%), whereas for *N* = 7 patients (46.7%), the removal of one or two teeth, in *N* = 2 patients (13.3%) the removal of three or four teeth, and in *N* = 3 patients (20%) the removal of five or more teeth was recommended. In the study group of patients who had been receiving dialysis for more than 3 years, in *N* = 14 patients (51.9%), no surgical treatment was necessary (Fig. 1). In *N* = 4 (14.8%) patients, one or two teeth, in *N* = 7 (25.9%) cases three or four teeth, and in *N* = 2 (7.4%) cases more than four teeth were recommended for extraction.

The difference of necessity for surgical interventions within the groups is statistically significant (*p* = 0.042). The necessity for surgical interventions after 2 or 3 years of dialysis compared to less than 2 years showed statistically significant differences (OR = 5.236; 95% CI: 1.244; 22.038; *p* = 0.024), whereas no statistically significant difference compared to more than 3 years of dialysis could be detected (OR = 1.235; 95% CI: 0.472; 3.233; *p* = 0.667). Several data are presented in Table 1.

**Table 1:** Illustration of surgical and conservative treatment need depending on duration of dialysis

	Duration of dialysis		
	< 2 years	2-3 years	> 3 years
No surgical treatment need	27 (57.4%)	3 (20%)	14 (51.9%)
Extraction of 1–2 teeth	13 (27.7%)	7 (46.7%)	4 (14.8%)
Extraction of 3–4 teeth	3 (6.4%)	2 (13.3%)	7 (25.9%)
Extraction of > 4 teeth	4 (8.5%)	3 (20%)	2 (7.4%)
Conservative treatment need	26 (55.3%)	11 (73.3%)	15 (55.6%)

### Conservative treatment need depending on dialysis duration

**Fig. 2:**
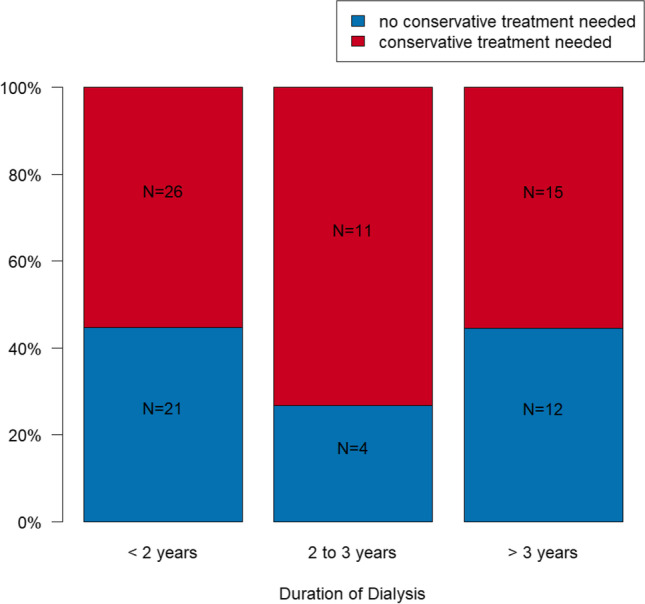
Conservative (red) and no conservative treatment (blue) need depending on duration of dialysis

Addressing the conservative treatment need (e.g., filling therapy, root canal treatment, root canal revision, or therapy of periodontitis) in patients with less than 2 years of dialysis, in *N* = 26 cases (55.3%), conservative treatment was necessary, whereas in the group with 2 or 3 years of dialysis, *N* = 11 cases (73.3%), and in the group with more than 3 years of dialysis, *N* = 15 (55.6%) showed conservative treatment need (Fig. 2). Conservative treatment need does not have a statistically significant relationship to duration of dialysis (*p* = 0.475). In this context, the necessity for conservative treatment after 2 or 3 years of dialysis compared to less than 2 years showed no statistically significant difference (OR=3.479; 95% CI: 0.827; 14.625; *p* = 0.089). Furthermore, no statistically significant difference compared to more than 3 years of dialysis could be detected (OR = 0.916; 95% CI: 0.338; 2.485; *p* = 0.864). Several data are presented in Table 1.

### Surgical treatment need depending on causative nephrological disease

**Fig. 3:**
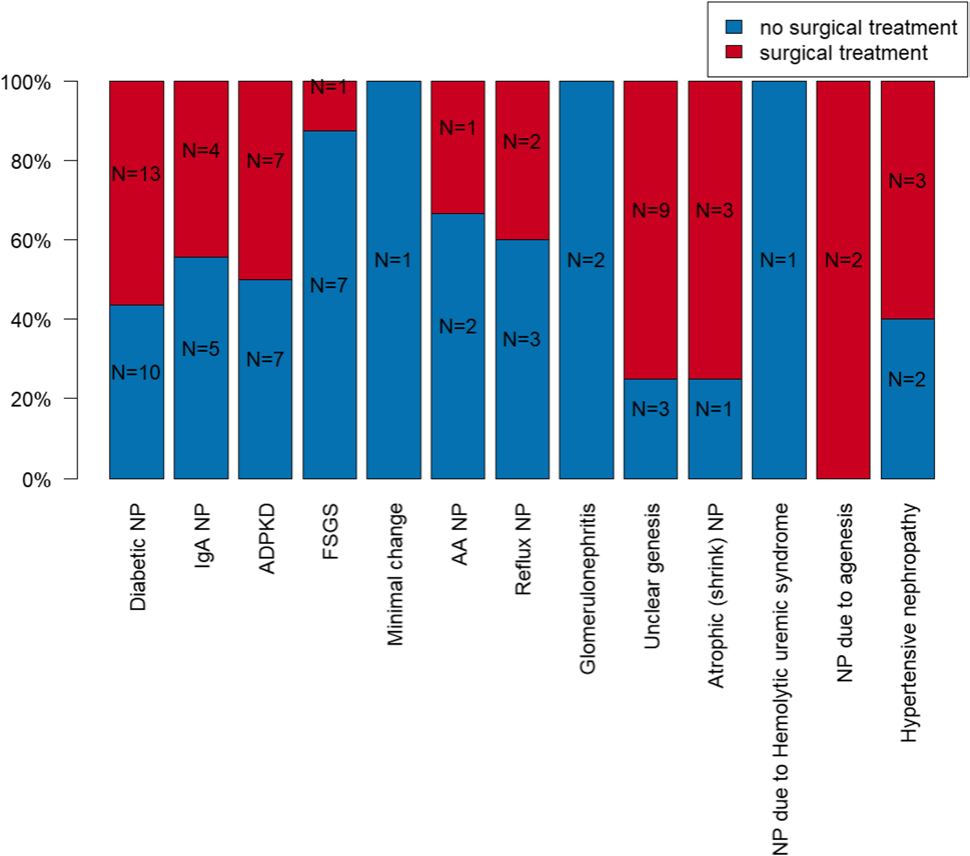
Illustration of relative and absolute patient numbers separated according to nephrological disease leading to dialysis who need (red) and who do not need (blue) surgical treatment prior to organ transplantation

Focusing on the necessity for surgical intervention depending on the causative nephrological disease, surgical intervention was necessary in *N* = 13 (56.5%) patients of the diabetic nephropathy group, in *N* = 9 (75.0%) patients of unclear nephropathy, in *N* = 7 (50.0%) patients with ADPKD, in *N* = 4 (44.4%) patients with IgA nephropathy, in *N* = 3 (60.0%) patients with hypertensive nephropathy, in *N* = 3 (75.0%) patients with atrophic (shrink) nephropathy, and in *N* = 2 patients of the vesicourethral reflux (40.0%), and in the nephropathy due to agenesis (100.0%) groups. Teeth also had to be removed in *N* = 1 patient (33.3%) of the amyloid nephropathy group and in the FSGS group (12.5%), whereas no surgical intervention was necessary in the minimal change nephropathy, other glomerulonephritis, and in the hemolytic-uremic syndrome groups. However, the causative nephrological disease has no statistically significant dependency on the necessity for surgical intervention (e.g., diabetes mellitus: OR= 1.048; 95% CI: 0.344; 3.197; *p* = 0.934). The comparison of surgical intervention need within the groups was not statistically significant (*p* = 0.187). Selected data are presented graphically in Fig. 3. Several data are presented in Table 2.

**Table 2:** Illustration of surgical and conservative treatment need depending on the causative nephropathy

	Surgical treatment need	Conservative treatment need
Diabetic nephropathy	*N* = 13 (56.5%)	*N* = 9 (39.1%)
Unclear nephropathy	*N* = 9 (75.0%)	*N* = 10 (83.3%)
ADPKD	*N* = 7 (50.0%)	*N* = 10 (71.4%)
IgA nephropathy	*N* = 4 (44.4%)	*N* = 2 (22.2%)
Hypertensive nephropathy	*N* = 3 (60.0%)	*N* = 3 (60.0%)
Atrophic (shrink) nephropathy	*N* = 3 (75.0%)	*N* = 3 (75.0%)
FSGS	*N* = 1 (12.5%)	*N* = 5 (62.5%)
Vesicourethral reflux	*N* = 2 (40.0%)	*N* = 3 (60.0%)
Nephropathy due to agenesis	*N* = 2 (100.0%)	*N* = 2 100.0%)
Amyloid nephropathy	*N* = 1 (33.3%)	*N* = 2 (66.7%)
Minimal change nephropathy	*N* = 0 (0.0%)	*N* = 1 100.0%)
Other glomerulonephritis	*N* = 0 (0.0%)	*N* = 2 100.0%)
Hemolytic-uremic syndrome	*N* = 0 (0.0%)	*N* = 0 (0.0%)

### Conservative treatment need depending on causative nephrological disease

**Fig. 4:**
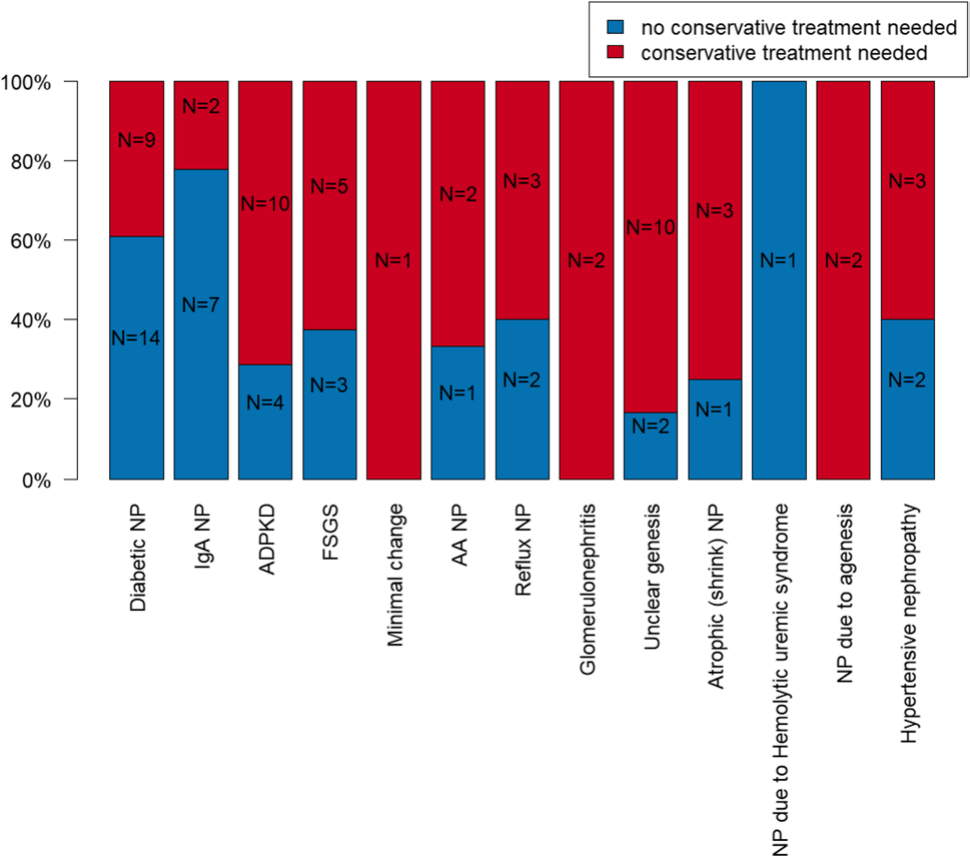
Illustration of relative and absolute patient numbers separated according to nephrological disease leading to dialysis who need (red) and who do not need (blue) conservative treatment prior to organ transplantation

Depending on the nephropathy necessitating dialysis, there were apparent differences among the groups. Conservative treatment was necessary in *N* = 10 patients in the ADPKD (71.4%) as well as in the unclear nephropathy (83.3%) groups. Conservative treatment was also needed in *N* = 9 (39.1%) patients in the diabetic nephropathy group, *N* = 5 (62.5%) in the FSGS group, and *N* = 3 patients in the vesicourethral reflux (60.0%) group, the hypertensive nephropathy (60.0%) group, and the atrophic (shrink) nephropathy (75.0%) group. Conservative treatment was also necessary in the IgA nephropathy (22.2%), amyloid nephropathy (66.7%), other glomerulonephritis (100.0%), and nephropathy due to agenesis (100.0%) groups with *N* = 2 patients each. Filling therapy was necessary in the one patient with minimal change nephropathy, whereas no treatment was indicated in the patients of the hemolytic-uremic syndrome group. The comparison of conservative intervention need within the groups was not statistically significant (*p* = 0.092). Selected data are presented graphically in Fig. 4. Several data are presented in Table 2.

### Surgical treatment need depending on alcohol/nicotine/drug abuse and age

Neither former (surgical treatment: OR= 0.741; 95% CI: 0.220; 2.496; *p* = 0.629; conservative treatments: OR= 0.686; 95% CI: 0.196; 2.394; *p* = 0.554) nor current (surgical treatment: OR= 0.882; 95% CI: 0.305; 2.556; *p* = 0.818; conservative treatments: OR= 0.682; 95% CI: 0.231; 2.013; *p* = 0.488) nicotine/alcohol or drug abuse cases showed statistically significant increased treatment need. Furthermore, patient age also showed no statistically increased surgical (OR= 1.019; 95% CI: 0.983; 1.056; *p* = 0.303) or conservative (OR= 1.003; 95% CI: 0.968; 1.040; *p* = 0.854) treatment necessity.

## Discussion

The therapy and surgical rehabilitation of patients suffering from terminal renal insufficiency, in the evaluation for potential transplant listing, presents a challenge to the oral and maxillofacial surgeon because the underlying nephropathy, comorbidities, and their associated medical treatment, as well as frequent/regular appointments for dialysis (two to three times per week) have to be integrated into the patient’s diagnosis, therapy, and aftercare protocol.

In this particular patient population, the scrupulous assessment of oral health status is of great importance for their further medical treatment, since it informs basic decisions regarding their care. A number of studies demonstrate that oral health and oral hygiene in patients suffering from terminal renal insufficiency are compromised [5, 10–12]. It has been clearly established that the onset, severity, and progress of periodontitis are associated with chronic renal disease [3]. This is especially true in patients with diabetic nephropathy, for whom the presence of deeper periodontal pockets [13], greater candida growth, and a greater amount of dental plaque could be demonstrated [14]. There is ample research describing the dental status of patients with chronic renal insufficiency [5, 15, 16]. Plaque index, calculus index, probing depth, and attachment loss are significantly higher in patients undergoing hemodialysis [15] encouraged by the lack of use of appropriate measures of oral hygiene [4] or preventive services [5]. Nevertheless, alongside a high proportion of missing teeth, a relatively low level of caries could be detected [16]. In spite of the obvious advantages of undergoing surgical and conservative rehabilitation of dental health status in the phase of dialytic treatment, as well as prior to receiving a kidney transplant, there is limited quantitative research with a focus on the number of inflammatory lesions, destroyed teeth, and/or residual roots with the requirement for surgical therapy.

Ruokonen et al. were able to show that dental status, age, and the presence of diabetes mellitus were independent risk factors for death [17]. They show in their research that deceased patients had fewer teeth and presented with a greater degree of oral infection [17]. Furthermore, it is biologically plausible that poor oral health would lead to adverse outcomes in patients with chronic renal disease, since they tend to display endothelial dysfunction [18], atherosclerosis [19], thrombosis [20], vascular injury and endotoxinemia [21], and chronic inflammation [22, 23].

Oral inflammatory lesions with the potential for transformation into life-threatening abscesses are more likely to develop in an adverse fashion in immunosuppressed patients. In light of the frequent necessity to pause the administration of immunosuppressive medications in order to adequately treat such abscess formations, and thereby raising the probability of transplant loss and even death, more research regarding the presence, development, and treatment of oral inflammatory lesions in patients with chronic kidney disease is an important step in the improvement of patient care.

Many publications addressing this topic utilize the DMFT index for assessing oral health status or the related quality of life [6, 14, 24]. However, this index contains no direct information about the necessity for surgical intervention. While the DMFT index expresses whether a given dentition required intervention in the past or present, it does not indicate whether this intervention was successfully undertaken or whether the pathological state persists.

What is at stake in improving the assessment of patients considered for medical immunosuppression are methods of evaluation that directly and more adequately lead to decisions for specific surgical and conservative treatment. Systematic studies specifically tailored toward informing therapeutic intervention are, however, rare [4].

The motivation for conducting the present study was to contribute to the knowledge base about this patient population. Particularly, we intended to analyze the frequency of necessity for surgical/conservative intervention in these patients depending on the duration of their dialysis treatment and the underlying nephropathy. We expected to find a relationship between the duration of dialytic treatment and the need for surgical intervention. What we found, though, was that patients with a 2- to 3-year history of dialysis displayed the highest and statistically significant need for surgical intervention, followed by patients with more than 3 years of dialysis and patients with less than 2 years of dialysis. The relatively low need for surgical therapy at the early stages of dialysis might reflect that patients in their first 2 years of dialysis still benefit from their pre-dialysis oral hygiene habits (e.g., regular attendance of a dental practice with a conservative treatment regimen consisting of fillings, professional dental cleansings, and regular feedback regarding personal success and techniques in carrying out oral hygiene measures), which may weaken over time as the manifestations of chronic kidney disease come to bear more prominently in the patient’s life. It may be that the concomitant decline in oral hygiene takes 2 to 3 years to reach a stage where conservative or surgical intervention becomes necessary, as our data showed the surgical treatment need to be at 80.0% and the conservative treatment need at 73.3% in the 2 to 3 years on dialysis group. Dialysis treatment is extremely physically and mentally exhausting, because of its frequency (usually three times per week) and length (several hours per individual session). This promotes circumstances where an individual’s daily life is dominated by dialysis, and other necessities, like formerly periodic visits to the dentist, which are seemingly no longer of primary concern for the maintenance of health and life, are neglected. Additional negative factors befalling this particular patient population, like malnutrition and negative changes of oral physiology due to dialysis (e.g., hyposalivation), can further accelerate the decline in oral health and exacerbate the problem. The group with the longest dialysis experience seems best adjusted to the limitations of the dialysis situation and its weekly rhythm, which is reflected in an improvement in oral hygiene (conservative treatment need: 55.6%, surgical treatment need 48.1%).

Great differences concerning the surgical treatment need and the underlying nephropathies/comorbidities, patient age, and former/current nicotine/alcohol/drug use were anticipated. However, our results showed no statistically significant relationship of the mentioned factors with the detected surgical/conservative treatment need. Especially in the patient group suffering from diabetic nephropathy, we anticipated greater surgical treatment need, since it is well known that diabetes can negatively impact oral health [13, 14].

Based on these results, the performed study shows that the need for surgical treatment in patients on dialysis due to terminal renal insufficiency is very inhomogeneous. In particular, the duration of dialysis (2–3 years) represents a potential factor driving an increased need for surgical and conservative treatment need.

A possible limitation of the study is the inhomogeneous size of the different study populations. However, group sizes are based on the prevalence of the etiology of renal failure.

## Conclusion

In the evaluation of the dental status, potential candidates for kidney transplantation in the second to third years of dialysis group show the greatest need for surgical and conservative intervention.
